# Adolescent self-harm and suicidal thoughts in the ALSPAC cohort: a self-report survey in England

**DOI:** 10.1186/1471-244X-12-69

**Published:** 2012-06-27

**Authors:** Judi Kidger, Jon Heron, Glyn Lewis, Jonathan Evans, David Gunnell

**Affiliations:** 1 School of Social and Community Medicine, University of Bristol, Canynge Hall, 39, Whatley Road, Bristol, BS8 2PS, UK

**Keywords:** Self-harm, Suicidal thoughts, Suicidal plans, Adolescence

## Abstract

**Background:**

Substantial numbers of adolescents self-harm, but the majority of cases do not reach the attention of medical services, making community studies essential. The prevalence of suicidal thoughts and plans at this age, and the inter-relationships between suicidal thoughts, plans and self-harm remain largely unexplored.

**Method:**

Cross-sectional analysis of self-reported questionnaire data collected from members of the Avon Longitudinal Study of Parents and Children (ALSPAC) birth cohort, England. Respondents (n = 4810) were aged 16–17 years old and have been followed up since birth.

**Results:**

Altogether 905 (18.8%) respondents had ever self-harmed. The prevalence of lifetime self-harm was higher in females (25.6%) than males (9.1%). The most commonly used method was self-cutting: this was used alone or in combination in 73.5% of episodes, compared to 10.0% who took overdoses alone or in combination with other methods. Of those who reported self-harm, 25.3% wanted to die during the most recent episode. Concurrent depression was associated with a greatly increased risk of self-harm (OR 5.43). Only 12.4% of participants sought medical help following their most recent episode of self-harm, although this figure was higher (30.1%) where self-harm was carried out with desire to die. Of the whole sample, 15.8% had ever thought of killing themselves, and 4.3% had ever made plans to kill themselves. Compared to those who had never self-harmed, those who had self-harmed but not wanted to die during the most recent episode were at increased risk of ever having had suicidal thoughts (37.6% compared to 7.8% χ^2^ =102.3, p < 0.001) and ever making suicidal plans (8.7% compared to 0.7%, χ^2^ =166.9, p < 0.001). As the frequency of self-harm increased, so did the risk of suicidal thoughts and plans.

**Conclusions:**

Self-harm and suicidal thoughts are common among 16/17 year olds. Although the majority of self-harm behaviour is not accompanied by a desire to die, all self-harm regardless of motivation is associated with increased risk of suicidal thoughts and plans, particularly when it is carried out repeatedly.

## Background

Self-harm is of public health concern not only because of the immediate physical harm that it causes, but also due to its association with psychological distress, and elevated risk of suicide [1,2]. It is rare under the age of twelve, but its incidence increases rapidly throughout the early teenage years, particularly among girls [3]. Most episodes do not come to the attention of healthcare services - in a recent UK sample, only one in eight 15–16 year olds received medical attention the last time they self-harmed [4] - therefore community-based studies are needed when estimating prevalence among teenagers. The largest such study in England to date - a self-report survey conducted in 41 schools - reported a lifetime prevalence of 13.2% among 15–16 year olds in 2000/2001 [4]. A more recent study in Sweden reported a lifetime prevalence of 17.1% among 17 year olds [5].

Several studies have distinguished between self-harm with intent to die (attempted suicide), and self-harm with no suicidal intent, commonly termed non-suicidal self-injury (NSSI) [6,7]. Rates of NSSI among community samples of adolescents are considerably higher than self-harm with suicidal intent: rates for the former have been found to be between 15.0%-21.2% [8-11], whereas suicidal attempts range from 4.0% to 10.5% [9,10,12]. However, the extent to which these represent separate behaviours, with different risk and protective factors and serving different functions, as opposed to more or less extreme variations of the same behaviour remains unclear [8], particularly as those who engage in NSSI are at increased risk of suicide attempts compared to those who do not self-harm at all [2,7].

The small number of studies that have examined suicidal thoughts and plans separately from actual self-harm report prevalences of approximately 11-17% for thoughts of killing oneself, and 9.5% for plans to do so [13,14]. Although self-harm is a known risk factor for future suicide [1], only two small community-based studies have examined the association between suicidal thoughts and self-harm with and without intent to die. Both found that those who engage in NSSI – the majority of adolescent self-harmers – are at a substantially higher risk of suicidal thoughts compared to those who have never self-harmed [2,9,10].

The only previous community-based study of teenage self-harm in England was conducted eight years before the present study [4]. Hawton et al. examined prevalence of self-harm and thoughts of self-harm, but did not include questions regarding thoughts or plans to kill oneself [15], therefore they were not able to examine the relationship between self-harm and suicidal thoughts and plans. Based on more recent data collected from a population-based birth cohort once participants were 16/17 years old – the Avon Longitudinal Study of Parents and Children (ALSPAC) [16] - this paper examines the prevalence and inter-relationships between self-harm with and without a desire to die, suicidal thoughts and suicidal plans among this age group. Specifically these questions are addressed:

· What is the prevalence of lifetime self-harm, suicidal thoughts and suicidal plans?

· What is the nature of the self-harm in terms of behaviour, motivation and consequences?

· What proportion of self-harm is motivated by a desire to die, and to what extent is self-harm, even where there is no desire to die, associated with suicidal thoughts and plans?

## Methods

### Sample

The Avon Longitudinal Study of Parents and Children (ALSPAC) (http://www.alspac.bris.ac.uk) is an ongoing population-based study investigating the effect of a wide range of environmental and other influences on the health and development of children [16]. Pregnant women resident in the former Avon Health Authority (which included the city of Bristol), in south-west England, who had an estimated date of delivery between 1 April 1991 and 31 December 1992, were invited to take part, resulting in a cohort of 14 541 pregnancies, 13,796 of whom were singletons or first-born of twins, and who were alive at one year of age. The current manuscript is focussed on the remaining offspring who received the questionnaire aged 16 (n = 9,384), that is they were not lost to follow up. Additional file 1: Appendix A shows the numbers present at each stage. The former County of Avon includes both urban and rural areas, and the extent to which the cohort is representative of children in the UK is discussed elsewhere [16]. The parents have completed regular postal questionnaires from pregnancy onwards, and the children have completed questionnaires from approximately 5 years.

### Outcome measures

Questions about self-harm and suicidal thoughts were included in a self-completion postal questionnaire, sent to study participants when they were aged 16 years (see Additional file 2: Appendix B for the full list of self-harm questions asked). Participants were asked “have you ever hurt yourself on purpose in any way (e.g. by taking an overdose of pills or by cutting yourself)?”, wording which was used in the Childhood Interview for DSM-IV Borderline Personality Disorder (CI-BDP) [17], asked during clinic interviews with the ALSPAC sample aged 11. Those who answered yes were asked further closed response questions regarding frequency (once, 2–5 times. 6–10 times, >10 times), what they did the last time they hurt themselves on purpose (4 response categories) and why they did it that time (6 response categories) - response options were a modified version of those in the CASE questionnaire [18]. Participants were also asked whether they sought medical help and how they felt following the most recent time, and whether they had ever seriously wanted to kill themselves when self-harming. The whole sample were then asked whether they had ever felt life was not worth living, wished they were dead and away from it all, thought of killing themselves, or made plans to kill themselves; questions which were drawn from a study of suicidal feelings in the USA [19].

Those who selected ‘other’ for what they did when they self-harmed and why they did it were invited to give further details (see Additional file 2: Appendix B). These free text responses were independently coded by JK and DG, based on the themes emerging from the data [20], for example ‘head butting a wall’ and ‘pulling hair’ were classified into the theme ‘self-battery’ for what was done, and ‘because I was grieving and it made me feel better’ was classified as ‘response to difficulty’ for why it was done. Where appropriate, themes arising from our data were classified using the categories from Hawton et al. in their coding of open responses [15]. Where more than one code applied, the response was given as many codes as needed. If the raters did not deem a described action to be self-harm then it was given a code of ‘not self-harm’. All cases that had received discrepant codes were examined and a consensus reached for each. Initial inter-rater agreement was 88% for ‘what they did’ and 72% for ‘why they did it’, but consensus was easily reached once the final coding frame was agreed. The coding frames and discrepant cases were then discussed with JE and GL, and a final coding frame and codes for each case agreed. All categories that emerged in our study had equivalents in Hawton et al’s study [15]; there were no discrepancies. In total nine out of 147 free text responses concerning method/reason for self-harm were excluded from the self-harm group following this process (this included six responses that referred to not eating).

### Demographic data

Demographic data, collected in previous ALSPAC questionnaires, were included in the analysis, to enable comparison of responders and non-responders, and to identify key psychosocial characteristics associated with self-harm. The following variables were used: i) mother’s social class (manual/intermediate/non-manual), ii) mother’s highest educational level (A level or ‘advanced level’ which are post compulsory schooling qualifications or higher education degree/O level or ‘ordinary level’ which are now defunct examinations taken at the end of compulsory schooling by students deemed more academically able/lower than O level which includes any other qualifications of a lower academic standard or no qualifications at all) as reported when the participant was first born, iii) mother’s score on the Edinburgh Postnatal Depression Scale (EPDS) (which has been validated for use in non-postnatal women [21], and was introduced in ALSPAC during pregnancy then retained for consistency) - using the cut-off point of 12/13 recommended for identifying a depressive illness [22] - when the participant was 11 years old, iv) participant’s gender, v) participant’s ethnicity (white/non-white) as reported by the mother prior to birth, vi) participant’s performance in national examinations undertaken aged 15/16 years in the final year of compulsory schooling (GCSEs/GNVQs) gained from national records (only available for those attending state schools), and vii) participant’s score on the short Moods and Feelings Questionnaire (SMFQ), included within the same questionnaire as the self-harm data. Following Patton et al., a score of 11 or more on the SMFQ was taken as indicative of depressive symptoms [23].

### Ethical approval

Ethical approval was obtained from ALSPAC’s Law and Ethics Committee, a registered Institutional Review Board and LREC. Information was included at the end of the questionnaire about help sources that participants could contact. Due to ALSAC’s rigorous anonymity rules, the research team could not follow up any participants with regard to their responses. However, participants could request on the questionnaire that a member of the ALSPAC team contact them with regard to the issues raised.

### Statistical analysis

The association between rates of self-harm and demographic data was assessed using χ^2^ statistical tests and univariable logistic regression models. As information on self-harm was not available for the whole ALSPAC cohort (due to those who were lost to follow up, and those who received the questionnaire but did not return it) there was the potential for bias both in the estimated prevalence and also in the association with demographic factors. This was explored using a multiple imputation, more details of which can be found in the section below. A secondary analysis looked in more detail at the reported cases of self-harm. The proportion of respondents who fell into each category for frequency of self-harm, reasons for it, and consequences in terms of subsequent feelings and the need to seek medical help were tabulated overall, and comparisons were made by gender using χ^2^ statistical tests. The characteristics of those who self-harmed with desire to die and those who self-harmed without desire to die the most recent time were compared using χ^2^ statistical tests. Finally, prevalence of suicidal thoughts and plans were examined across the whole sample, comparing those who had ever self-harmed with those who had not, again using χ^2^ statistical tests. This second set of analyses did not involve data imputation as respondents typically had either all or none of the measures of interest. All analyses were carried out using Stata version 10.0.

### Missing data imputation

We assessed the impact of non-response and missing data on our findings using MICE (Multivariate Imputation by Chained Equations) [24] implemented using the *ice* routine [25] in Stata. This procedure creates multiple copies of the dataset and in each dataset replaces missing data with imputed values, sampled from their predictive distribution [26]. The use of this method is based on the Missing At Random (MAR) assumption, namely that conditional on the other data included in the imputation model, there should not be systematic differences between observed and missing values for a given variable. A number of variables were included to assist with the imputation. These included indicators of family adversity at enrolment such as home overcrowding, financial problems, and lack of social support; earlier (more complete) measures of the predictive factors considered in this manuscript such as mother’s social class, and other measures more proximal to the outcome such as maternal and young person’s substance use behaviour in early adolescence, and reported self-harm from an earlier clinic at 11 years. Missing data for the binary measure of self-harm was imputed using logistic models and additional data on demographics employed binary and multinomial logistic models as appropriate. 100 imputed datasets were derived, each entailing 20 cycles of regression switching.

Imputation was carried out on two different samples: i) to estimate values for those among the sample of 4,810 who had information of self-harm but an incomplete set of the demographic predictors described above and ii) to impute missing data for the 4,529 participants (32.8% of the initial cohort) that were sent the relevant questionnaire but did not return it to give a useable sample of 9,268 to enable us to estimate prevalence amongst the cohort members who were sent questionnaires.

It has been suggested that there may be little benefit to imputing data for individuals who lack outcome data as their inclusion in one’s regression model may add nothing but noise [27]. To address this as far as possible we included auxiliary variables strongly related to the outcome (as described above), and we paid close attention to the Monte Carlo error associated with both regression parameters and their standard errors.

## Results

Of the 9384 participants that received the questionnaire, 4855 returned it (51.7%) and 4810 (51.3%) responded to the self-harm questions. The mean age of respondents at the time the questionnaire was completed was 16 years and 8 months (standard deviation (SD) 2.9 months). Those who returned the questionnaire were more likely than non-respondents to be female, have a mother in a non-manual social class, and to have relatively high educational qualifications (see Table 1 for more details).

**Table 1:** Comparison of responders and non-responders by key background variables

		**Remain in study but non-response to the self-harm questionnaire N(%)**	**Self-harm data available N(%)**	**χ**^**2**^	**p-value**
Gender	Males	2,640 (58.3%)	1,997 (41.1%)	276.0	<0.001
	Females	1,889 (41.7%)	2,858 (58.9%)		
Mother’s social class	Prof/managerial	1,137 (33.7%)	1,855 (45.0%)	103.4	<0.001
	Intermediate	1,814 (53.8%)	1,895 (46.0%)		
	Manual	420 (12.5%)	370 (9.0%)		
Mother’s highest educational qualification	A level/degree	1,243 (30.0%)	2,272 (48.1%)	367.0	<0.001
	O-level	1,543 (37.2%)	1,569 (33.2%)		
	LT O-level	1,364 (32.9%)	886 (18.7%)		
Ethnicity	White	4,223 (94.4%)	4,630 (95.9%)	12.8	<0.001
	Non-white	253 (5.7%)	196 (4.1%)		
GCSE/GNVQs at grades A*-C	5 or more	2,120 (52.8%)	3,373 (80.8%)	727.0	<0.001
	Less than 5	1,898 (47.2%)	803 (19.2%)		
Mother’s EPDS score when child aged 11	Less than 13	2,177 (86.2%)	3,749 (89.1%)	12.7	<0.001
	13 or more	350 (13.9%)	460 (10.3%)		

The total number of respondents who had ever self-harmed was 905 (18.8%, 95% CI 17.7% to 19.9%). Prevalence was higher in females (25.6%) than males (9.1%) and overall prevalence was not substantially different in imputed models taking account of missing data due to questionnaire non-response - the estimated lifetime prevalence for self-harm was 18.4% (95% CIs 17.3%-19.6%) (see Additional file 3).

There was strong evidence of an association (p < 0.001) between self-harm and both gender and current negative mood (see Table 2). Females were at almost 3.5 times more likely to report self-harm than males and individuals with negative mood symptoms were more than 5 times greater risk. The risk of self-harm was also somewhat higher amongst among respondents whose mother scored 13+ on the EPDS five years earlier (OR = 1.48 [1.17, 1.86], p = 0.001), those whose mothers were of manual social class (OR = 1.46 [1.12, 1.90], p = 0.005) and those achieving grades A*-C in less than five GCSE/GNVQ exams (1.20 [1.03, 1.34], p = 0.025). Finally there was no strong evidence of an association between self-harm and either maternal education or the young person’s ethnicity. Comparison of these results showed good agreement across the two imputation samples considered and we were confident that the estimates were not being overly impacted by noise due to imputation of the outcome (see Additional file 3).

**Table 2:** **Association of ever having self-harmed at age 16 with key demographic variables**^**1,2**^

		**Ever self-harmed**^**3**^		
		**N (%)**	**OR [95% CI]**	**p-value**
Gender	Males	180 (9.1%)	1.00 ref	<0.001
	Females	725 (25.6%)	3.42 [2.87, 4.07]	
Mother’s social class^3^	Prof/managerial	339 (18.4%)	1.00 ref	0.004
	Intermediate	323 (17.2%)	0.92 [0.77, 1.08]	
	Manual	91 (24.8%)	1.46 [1.12, 1.90]	
Mother’s highest educational qualification	A level/degree	400 (17.7%)	1.00 ref	0.114
	O-level	317 (20.4%)	1.19 [1.01, 1.40]	
	< O-level	162 (18.5%)	1.06 [0.86, 1.29]	
Ethnicity	White	865 (18.9%)	1.00 ref	0.225
	Non-white	30 (15.5%)	0.79 [0.53, 1.17]	
GCSE/GNVQs at grades A*-C^4^	5 or more	613 (18.3%)	1.00 ref	0.027
	Less than 5	171 (21.8%)	1.20 [1.03, 1.34]	
SMFQ score aged 16	Less than 11	512 (13.2%)	1.00 ref	<0.001
	11 or more	379 (45.3%)	5.43 [4.60, 6.40]	
Mother’s EPDS score when child aged 11	Less than 13	655 (17.6%)	1.00 ref	0.001
	13 or more	109 (21.0%)	1.48 [1.17, 1.86]	

Notes

1. Logistic regression based on available data (n < = 4810).

2. Number of respondents with missing data was 0 for gender, 735 for mother’s class, 128 for mother’s highest educational qualification, 29 for ethnicity, 679 for own educational qualifications, 116 for SMFQ score, and 646 for mother’s EPDS score.

3. Non-respondents to “have you ever hurt yourself on purpose?” = 45.

4. Where the more A*-C grades obtained the better.

**Figure 1:**
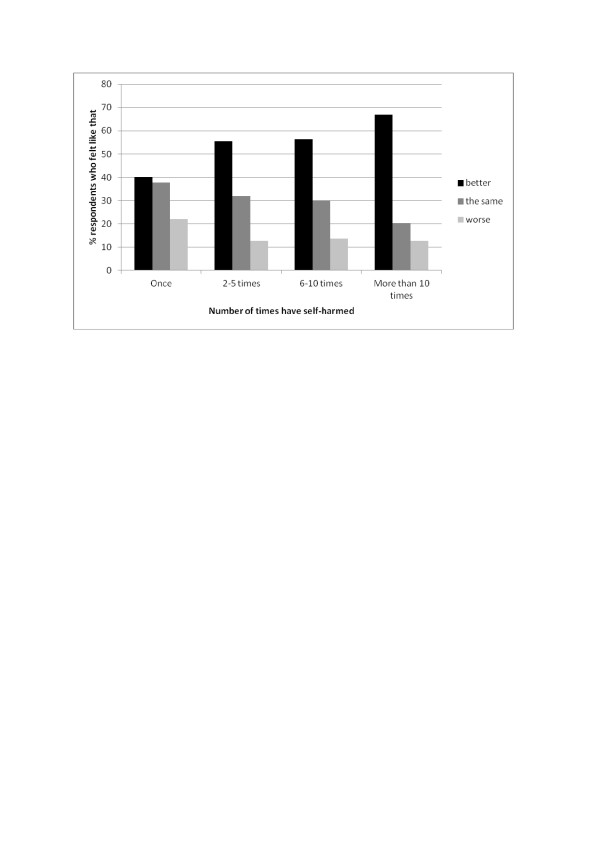
Association between frequency of self-harm and how respondent felt subsequently.

Table 3 gives details of frequency, actions and consequences of self-harm behaviour. Just under one third of those who had ever self-harmed, which equated to 5.7% of the total sample, said that they had ever seriously wanted to kill themselves while self-harming, with no gender difference. A majority of those who had self-harmed had done so in the past year, 7.8% in the past week. The single method most often used the last time respondents self-harmed was cutting for both genders (64.1%). This was followed by self-battery (8.3%) - which included actions such as biting, pulling hair and head butting walls - while overdosing (4.1%) and burning oneself (1.9%) were less common methods. Just over 20% engaged in more than one method of self-harm at once. Only 12.4% of respondents had sought medical help following the most recent self-harm act, with no gender difference. More than half of those who had self-harmed had done so more than once in the past year, and 25.3% had done so 6 or more times. Approximately half felt better after self-harming the most recent time, with boys more likely to feel neither better nor worse compared to girls. As the frequency of self-harm increased, so did the likelihood that the person would feel better subsequently (see Figure 1). Compared to those who had self-harmed once, those who had self-harmed more than 10 times were three times as likely to feel better rather than worse or the same following self-harm (OR = 3.02, 95%CI: 1.93-4.72), (p value for trend across categories = <0.001).

**Table 3:** Description of self-harm actions and consequences among the 905 participants who reported self-harm

	**N (% of those who have ever self-harmed)**					
		**Males ** **(n = 180)**	**Females ** **(n = 725)**	**TOTAL ** **(n = 905)**	**χ**^**2**^	**p-value**
Ever seriously wanted to kill self during self-harm act^1^	Yes	54 (30.3)	222 (31.0)	276 (30.9)	0.03	0.863
	No	124 (70.0)	494 (69.0)	618 (69.1)		
Timing of most recent episode^2^	Within the past week	12 (6.8)	58 (8.1)	70 (7.8)	0.4	0.799
	More than a week but within a year	93 (52.8)	364 (50.6)	457 (51.1)		
	More than one year ago	71 (40.3)	297 (41.3)	368 (41.1)		
What they did (most recent time)^3^	Swallowed pills only	1 (0.6)	36 (5.0)	37 (4.1)	46.6	<0.001
	Self-cut only	105 (58.7)	475 (65.7)	580 (64.1)		
	Burnt themselves only	7 (3.9)	10 (1.4)	17 (1.9)		
	Self-battery only	33 (18.4)	42 (5.8)	75 (8.3)		
	Other single method^7^	3 (1.7)	7 (1.0)	10 (1.1)		
	Cut and overdose	4 (2.2)	49 (6.8)	53 (5.9)		
	Cut and self-battery	5 (2.8)	27 (3.7)	32 (3.5)		
	Other multiple method^8^	22 (12.2)	79 (10.9)	98 (10.9)		
Sought medical help (most recent time)^4^	Yes	19 (10.6)	93 (12.8)	112 (12.4)	0.6	0.456
	No	161 (89.4)	632 (87.2)	793 (87.6)		
Frequency of self-harm in the last year^5^	Once	25 (23.8)	97 (23.1)	122 (23.2)	3.1	0.377
	2-5 times	48 (45.1)	171 (40.7)	219 (41.7)		
	6-10 times	9 (8.6)	60 (14.3)	69 (13.1)		
	More than 10 times	23 (21.9)	92 (21.9)	115 (21.9)		
How they felt after the most recent episode of self-harm)^6^	Better than before	77 (44.0)	366 (50.8)	443 (49.4)	16.5	<0.001
	The same	79 (45.1)	217 (30.1)	296 (33.0)		
	Worse than before	19 (10.9)	138 (19.1)	157 (17.5)		

Notes

1. Non-respondents = 11.

2. Non-respondents = 10.

3. Non-respondents = 3.

4. Non-respondents = 0.

5. As this question did not include ‘never’ as an option, this analysis was carried out only with those who did not select ‘more than a year ago’ in answer to when they last hurt themselves on purpose, n = 525.

6. Non-respondents = 9.

7. Excess of alcohol/drugs (n = 2), jumping/dangerous behaviour (n = 2), hanging/strangulation/suffocation (n = 1) or method not specified (n = 2).

8. Includes other combinations of two methods, and three or more methods of any combination.

**Figure 2:**
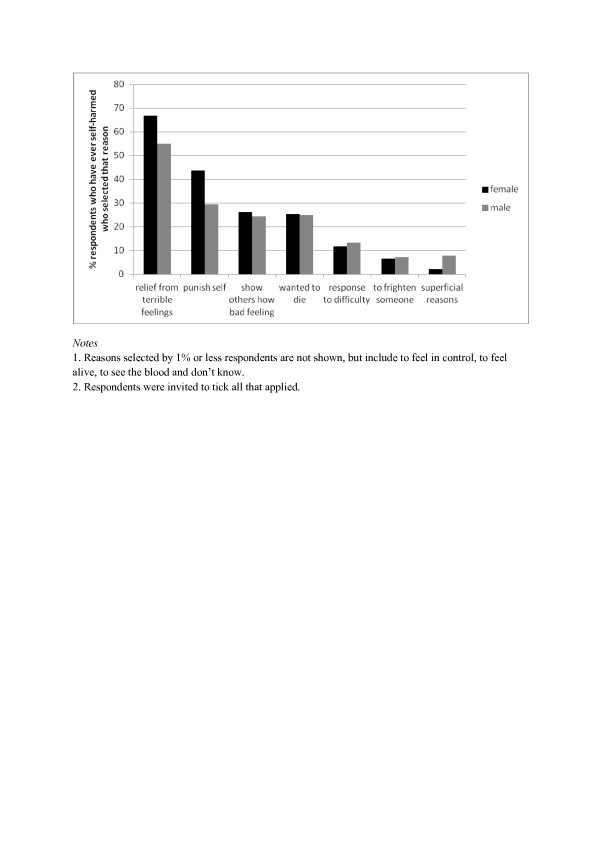
Reasons given for the most recent time participants self-harmed.

Figure 2 shows the reasons given for the most recent self-harm episode. The most common reasons were to gain relief from terrible feelings (64.4%) and a desire to punish oneself (41.0%). Of the whole sample, 25.3% gave ‘wanted to die’ as a reason (25% males and 25.4% females). Females were more likely than males to select desire to punish self (43.8% compared to 29.4%, χ^2^ = 12.30, p < 0.001) and to gain relief from terrible feelings (66.7% compared to 55.9%, χ^2^ = 8.56, p = 0.003), whereas males were more likely than females to choose ‘superficial reasons’ such as “I was curious” or “because my friend was” (7.8% compared to 2.2%, χ^2^ = 13.96, p < 0.001). There were no other large gender differences in reasons given.

Table 4 compares those who had wanted to die the most recent time they had self-harmed with those who had not given this as a reason. Those who wanted to die were much more likely to have poorer educational qualifications and a greater number of depressive symptoms on the SMFQ at age 16, and slightly more likely to have a mother in a manual social class at the time of their birth. This group were also much more likely to have taken an overdose the most recent time they self-harmed, whereas there was no difference between the groups regarding whether they had cut themselves. Those who had wanted to die were also less likely to feel better after the last episode of self-harm, and much more likely to have sought medical help (30.1% vs. 6.4%).

**Table 4:** **Comparison of those whose last episode of self-harm was associated with vs. without a desire to die: their characteristics, and the characteristics of the act and its consequences**^**1**^

	**Wanted to die (n = 229) N (%)**	**Did not want to die (n = 676) N (%)**	**χ**^**2**^	**p-value**
Female	184 (80.3)	541 (80.0)	0.01	0.917
Mother in manual social class	29 (12.6)	62 (9.2)	4.8	0.091
Less than 5 GCSEs/ GNVQs A*-C	77 (33.6)	94 (13.9)	42.4	<0.001
8 or more on SMFQ aged 16	196 (85.6)	410 (60.7)	48.1	<0.001
Took pills	72 (31.4)	49 (7.2)	86.4	<0.001
Cut self	189 (82.5)	569 (84.2)	0.3	0.561
Felt better subsequently	86 (37.9)	357 (53.4)	16.2	<0.001
Sought medical help afterwards	69 (30.1)	43 (6.4)	89.1	<0.001

Notes

1. Non-response ranged from 0 to 9 (how felt)

**Figure 3:**
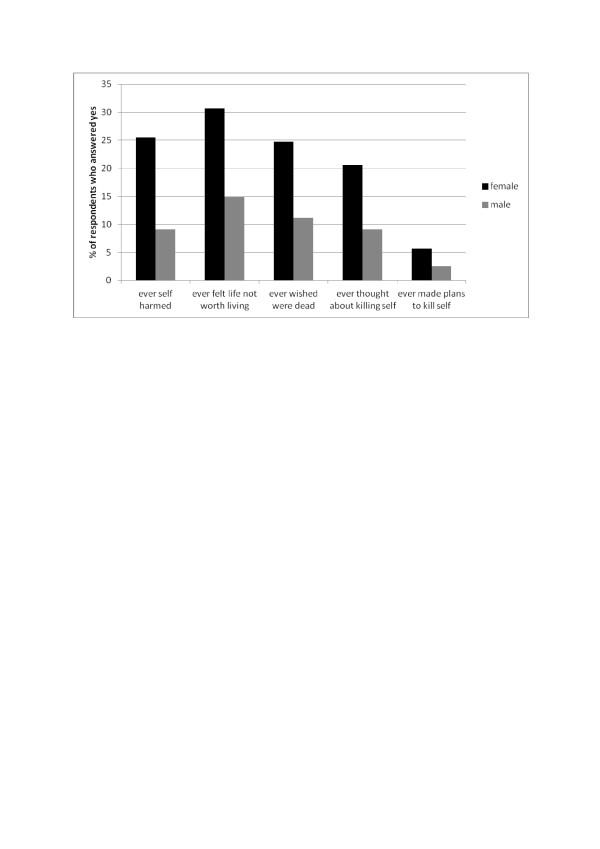
Percentage of respondents who have ever self harmed or experienced suicidal thoughts, by gender.

Table 5 shows the percentage of respondents who answered yes to the series of questions on suicidal thoughts and plans. Just under one quarter of all respondents had ever felt that life was not worth living, with 15.8% (95% CIs 14.5%-16.6%) having thought of killing themselves, and 4.3% ever having made plans to kill themselves (95% CIs 3.7-4.8). As shown in Figure 3, females were more than twice as likely as males to have ever self-harmed, felt life was not worth living, wished they were dead and away from it all, had thoughts of killing themselves and made plans to kill themselves (all χ^2^ test p values <0.001). Large differences in all the questions relating to suicidal thoughts and plans were visible between those who had self-harmed with desire to die the most recent time, those who had self-harmed without desire to die the most recent time and those who have never self-harmed (Table 5). Approximately 90% of those who had self-harmed with desire to die and over one third of those who had self-harmed without desire to die had ever thought of killing themselves, compared to 7.7% of the never self-harmed group. And over half of those who had self-harmed with desire to die had ever made plans to kill themselves, compared to 8.7% of those who had self-harmed without desire to die, and just 0.7% in the never self-harmed group.

**Table 5:** **Prevalence of suicidal thoughts/plans in those whose most recent episode of self-harm was associated with a desire to die, those who self-harmed with no desire to die, and those who have never self-harmed**^**1**^

	**Never self-harmed (n = 3,905) N (%)**	**Self-harmed did not want to die (n = 676) N (%)**	**Self-harmed wanted to die (n = 229) N (%)**	**Total N (%)**
Has ever felt that life is not worth living	597 (15.4)	338 (50.6)	220 (96.1)	1,155 (24.2)
Has ever wished was dead	425 (10.9)	279 (41.3)	215 (93.9)	919 (18.9)
Has ever thought of killing self	304 (7.8)	254 (37.6)	207 (90.4)	765 (15.8)
Has ever made plans to kill self	29 (0.7)	59 (8.7)	121 (52.8)	209 (4.3)

Notes

1. χ^2^ tests showed a strong association (p < 0.001) across the three groups for all the measures. A comparison of those who had never self-harmed with just those who had self-harmed but not wanted to die also showed a strong association (p < 0.001) for all measures

**Figure 4:**
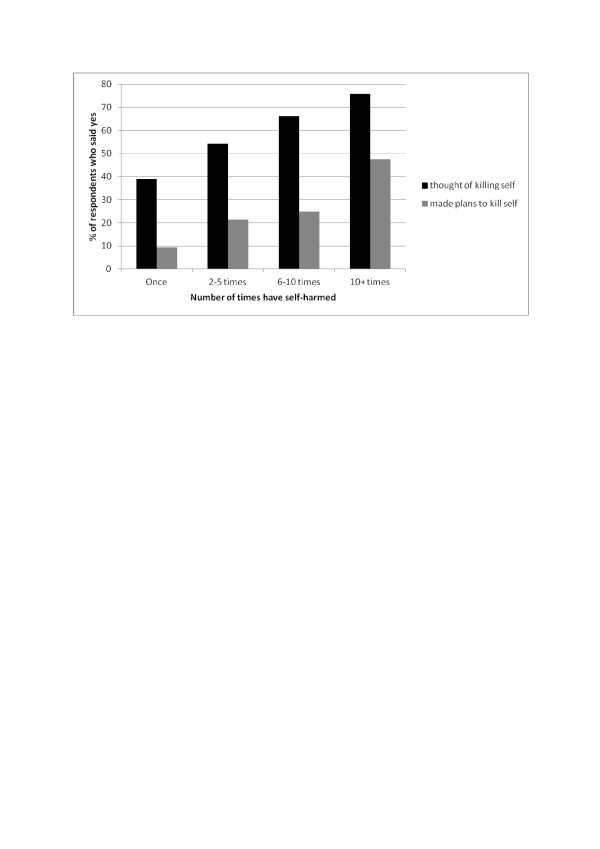
Comparison of self-harm with desire to die and self-harm with no desire to die the most recent time by background variables, and self-harm actions and consequences.

Figure 4 shows the relationship between frequency of self-harm and suicidal thoughts or plans. Compared to those who had self-harmed once, those who had self-harmed 2–5 times were nearly twice as likely to have thought of killing themselves (OR = 1.86, 95%CIs: 1.34-2.58), those who had self-harmed 6–10 times were more than three times as likely to have done (OR = 3.08, 95%CIs: 1.84, 5.17) and those who had self-harmed more than ten times were nearly five times as likely to have done (OR = 4.93, 95%CIs: 3.06, 7.94). Similarly, compared to those who had self-harmed once, those who had self-harmed 2–5 times were more likely to have made plans to kill themselves (OR: 2.59, 95%CIs: 1.61, 4.17), those who had self-harmed 6–10 times were more than three times more likely to have done (OR:3.18, 95%CIs: 1.69, 6.00) and those who had self-harmed more than 10 times were approximately eight times more likely to have done (OR: 8.64, 95%CIs: 5.12, 14.60).

## Discussion

### Main findings

Almost one in five (18.8%) 16–17 year olds in the ALSPAC cohort reported that they had self-harmed on one or more occasions, with an almost threefold higher prevalence in females than males. Altogether, 5.7% of responders had ever wanted to kill themselves while self-harming. Self-harm was more likely among those with higher depressive symptoms and lower educational qualifications, and those whose mother was in a manual social class and had suffered from depression. When the most recent episode of self-harm was considered, those whose self-harm was associated with a desire to die had higher levels of depressive symptoms and lower educational qualifications compared to those who self-harmed with no desire to die.

The majority who had ever self-harmed had done so at least twice in the last year, with one quarter having self-harmed 6 times or more in that time period. Self-cutting was the most common method in both genders, but girls were more likely to have taken an overdose compared to boys. Those who self-harmed with a desire to die were also more likely to have taken an overdose, compared to those who did not express a desire to die. A range of reasons were given for the most recent self-harm, the commonest being to gain relief from terrible feelings followed by a desire to punish oneself, with one quarter wanting to die. Just under half of those who had ever self-harmed felt better after the last time, although this percentage was lower among those who had wanted to die. Feeling better subsequently was associated linearly with frequency of self-harm; the increasing rewards in terms of positive affect may help explain why many individuals self-harm with greater and greater frequency. Nevertheless, those who frequently self-harmed were a high risk group and almost half had made plans to kill themselves at some point in their lives. Only 12% had ever sought medical help following an episode of self-harm; even amongst those who self-harmed with a desire to die during that episode only one third had sought medical help subsequently, indicating that a high number of suicide attempts are likely to remain hidden from medical services among this age group.

Just under a quarter of respondents had ever thought life was not worth living, approximately 19% had ever wished they were dead, 15% had ever thought of killing themselves and 4% had ever made plans to do so. Females were more likely to have experienced all these levels of suicidal thoughts compared to males. Unsurprisingly, those who had wanted to die the most recent time they self-harmed were the most likely to have thought about and made plans to kill themselves (90.4% and 52.8% respectively), but those who had self-harmed without desire to die the most recent time were at increased risk of suicidal thoughts and plans compared to those who had never self-harmed. The association between self-harm and suicidal thoughts was stronger the more frequent the self-harming behaviour.

### Strengths/limitations

This is the first UK-based birth cohort study to look at teenage self-harm and suicidal thoughts and plans, and one of the largest studies internationally to examine these interrelationships in detail. Our results confirm previous findings that much adolescent self-harm, even where there was a desire to die, does not receive medical attention, emphasising the importance of community-based studies for providing information about prevalence. A key advantage of this study is the size and representativeness of the sample at the outset and the detailed prospectively recorded measures of socio-economic circumstances, development and mental health. The loss to follow up over the years may have led to selection bias, as those from the original sample who did not receive the questionnaire were more likely to be male and non-white, to have less than five GCSE/GNVQs at grades A*-C, and to have a mother in a manual social class, with lower educational qualifications, and with depressive symptoms when the individual was aged 11. Further, those who received the questionnaire but did not return it differed from respondents in similar ways. The results from the imputation models indicate that the differences between respondents and non-responders did not have a large impact on prevalence figures for self-harm or risk factor associations. This may be because males were more likely to have missing data yet were less likely to self-harm, whereas those with low educational performance were more likely to have missing data and were more likely to self-harm, so these features of the missing data may have cancelled each other out.

The use of self-report may have encouraged more honest answers than an interview situation [28], although the stigma of self-harm may still have led to underreporting and therefore an underestimation of prevalence. ALSPAC has very strict procedures in terms of anonymity and confidentiality, with which study participants are familiar, which it is hoped will have minimised this possibility. A second problem with using self-report is that participants are defining self-harm for themselves. The free text responses regarding what they did gave some insight into the behaviours that participants included - of the 147 who gave such a response only 9 were excluded. However, for those who did not include a free text response, we were reliant on their own decisions as to what constituted self-harm. Relatedly, respondents who had depressed mood may have interpreted their own behaviour and underlying motivations in more negative ways, which may have introduced bias in the association seen between depressed mood and self-harm, particularly self-harm with intent to die. A third problem, relating to the face validity of the questionnaire, is that approximately 10% of those who had wanted to die during the most recent episode of self-harm had never had thoughts of killing themselves. This indicates that for some respondents ticking ‘I wanted to die’ as a reason for their most recent self-harm did not necessarily mean they were trying to kill themselves - it may be that for this group the self-harm was a reaction to the misery they were experiencing, but was not an attempt to fulfil their desire to die. Therefore the distinction made in this paper between those who wanted to die and those who did not during the most recent self-harm act, although a useful indication of the most serious cases of self-harm, is not exactly equivalent to a distinction between suicide attempts and NSSI.

### Relevance to wider literature

The prevalence of self-harm in ALSPAC is higher than that reported in the only other community-based study in England of similar size (13.2%) [4]. This may be partially explained by a number of methodological differences. Our sample was approximately one year older, and we had a higher proportion of girls (58.9% compared to 42.7% [7]), although this has been adjusted for in the imputation models, with little change to prevalence. Further, we used the term ‘hurt yourself on purpose’ – to remain consistent with questions asked of the ALSPAC sample aged 11 during clinic interviews - whereas Hawton et al. used ‘harm yourself’ which may have implied more serious self-harm and led to inclusion of fewer episodes. The earlier study collected data through schools which may have led to greater concerns about confidentiality and higher levels of under reporting, and also to an underestimation of prevalence due to absent pupils [4]. However, the increase in prevalence may also reflect a genuine rise in self-harm rates over time as the data were collected approximately eight years apart. There is some evidence that self-harm rates among teenagers and young adults have increased over recent decades so this rise may have continued [3,29]. Further, increases in psychological distress among teenagers in the UK – strongly associated with self-harm in this and other research [5] - have also been reported in some studies [30], although not in others [31].

The prevalence of suicidal thoughts and plans is similar to that in smaller studies from other countries [13,14], and indicates that thoughts of killing oneself are as common among teenagers as self-harm. Although the two are associated in this sample, the relationship is complex. The majority of self-harm behaviour appears to be non-suicidal in intent, with approximately three quarters not wanting to die the last time they self-harmed. Conversely, a minority of the sample had never self-harmed but had had suicidal thoughts, indicating that self-harm is not a necessary precursor to or outcome of suicidal thoughts. There was evidence that self-harm behaviour motivated by a desire to die was different from other self-harm, in terms of method used – taking an overdose – and consequences, in that the individual was less likely to feel better, and more likely to seek medical help. Such findings support the distinction between NSSI, which tends to refer to actions involving tissue damage such as self-cutting [2,6], and self-harm with suicidal intent, which is most closely associated with overdosing. However, a strong association was observed between self-harm without a desire to die the most recent time and suicidal thoughts and plans, with over a third having had suicidal thoughts, and approximately 9% having made plans to kill themselves. This resonates with previous findings that, although individuals who self-harm with suicidal intent are at the highest risk of suicidal thoughts and attempts, those who engage in NSSI are also at significantly higher risk than those who do not self-harm at all [2,6,9]. Further, this elevated risk of suicidal thoughts and plans increases as the self-harm becomes more frequent, thus those who self-harm the most frequently are potentially at the most risk for suicide [32]. The nature and direction of the relationship between self-harm and suicidal thoughts remains unclear. It may be that suicidal thoughts lead the individual to self-harm – either to enact or to reduce the urge [11] - or that self-harm causes psychological distress which then contributes to suicidal thoughts [33], or that the two are co-occurring phenomena caused by a third factor, for example low self-esteem. Furthermore, it has been speculated that NSSI may make suicide/suicidal self-harm more “accessible” to high risk individuals as suicide is seen as a frightening and extreme action, but repetitive engagement in NSSI may make people more courageous and prepared to make suicide attempts [34].

The gender difference in prevalence of self-harm is striking, and replicates other studies of this age group [4,18]. However, these extreme differences reduce in later periods of the life course, resulting in a female prevalence that is only slightly higher than the prevalence for males [35,36]. This suggests that the gap observed during adolescence may partly be due to males and females being at different developmental stages.

## Conclusions

Given the significant, possibly increasing, numbers of teenagers who self-harm, and the strong association with suicidal thoughts and plans not only among those who wish to die but also among those engaging in NSSI, more research is needed into the long-term health consequences for those who engage in this behaviour, most of whom do not receive medical help. More also needs to be known about the potentially different functions that self-harm serves, and the causal pathways that link self-harm and suicidal thoughts among this age group. Qualitative research in particular, which is ideally suited to gaining insights into the meanings and consequences of behaviours from the perspective of participants themselves, is currently lacking in the area of self-harm. One commonly held belief is that a form of non-suicidal, possibly habitual self-harm exists that is conceptualised as ‘attention seeking’ or ‘a cry for help’ and that somehow needs not attract as much concern as self-harm with more serious physical consequences or intent. Our finding that all self-harm is linked to increased risk of suicidal thoughts and plans, and that the more frequent the self-harm the greater the risk of suicidal thoughts and plans, calls such views into question.

Although self-harm is often conceptualised as a way of regulating difficult emotions [11], in this study only half of those who self-harmed without suicidal intent and just over one third of those who self-harmed and wanted to die felt better as a result, suggesting that for many the action has not brought relief, or has replaced one distressing feeling for another. Knowledge such as this may be a useful starting point for entering discussion with teenagers as to which strategies for regulating difficult emotions might be more effective. Once greater understanding is reached regarding the causes, functions, outcomes and long-term risks of self-harm for teenagers, community-based interventions can be developed to reduce its prevalence and support those who engage in such behaviours.

## Competing interests

The authors declare they have no competing interests.

## Authors’ contributions

JK co-conceived and designed the study, developed the self-harm questions, conducted the main data analysis and wrote the paper. JH advised on the ALSPAC dataset, contributed to the statistical analysis, conducted the missing data analysis, and co-wrote the methods and results sections. GL contributed to the study design, the self-harm questions and the data analysis and interpretation, and critically revised the manuscript. JE contributed to the data analysis and interpretation, and critically revised the manuscript. DG co-conceived and designed the study and self-harm questions, advised on data analysis, and contributed to the interpretation of findings and the writing of the paper. All authors read and approved the final manuscript.

## Pre-publication history

The pre-publication history for this paper can be accessed here:

http://www.biomedcentral.com/1471-244X/12/69/prepub

## Supplementary Material

Additional file 1 Flow Chart of Cohort Participants. Click here for file

Additional file 2 Questions on self-harm. Click here for file

Additional file 3 Comparison of relationship between self-harm (SH) and background variables for different imputation samples. Click here for file

## References

[B1] Hawton K Zahl D Suicide following deliberate self-harm: long-term follow-up of patients who presented to a general hospital Br J Psychiatry 2003 182 537 542 10.1192/bjp.182.6.537 12777346

[B2] Muehlenkamp JJ Guttierrez PM Risk for suicide attempts among adolescents who engage in non-suicidal self-injury Arch Suicide Res 2007 11 1 69 82 10.1080/13811110600992902 17178643

[B3] Hawton K Hall S Simkin S Bale E Bond A Deliberate self-harm in adolescents: a study of characteristics and trends in Oxford, 1990–2000 J Child Psychol Psychiatry 2003 44 8 1191 1198 10.1111/1469-7610.00200 14626459

[B4] Hawton K Rodham K Evans E Weatherall R Deliberate self-harm in adolescents: self-report survey in schools in England BMJ 2002 325 1207 1211 10.1136/bmj.325.7374.1207 12446536 PMC135492

[B5] Landstedt E Gillander Gadin K Deliberate self-harm and associated factors in 17-year-old Swedish students Scand J Public Health 2011 39 1 17 25 10.1177/1403494810382941 20846995

[B6] Nock MK Joiner TE Gordon KH Lloyd-Richardson E Prinstein MJ Non-suicidal self-injury among adolescents: Diagnostic correlates and relation to suicide attempts Psychiatry Res 2006 144 65 72 10.1016/j.psychres.2006.05.010 16887199

[B7] Wichstrom L Predictors of non-suicidal self-injury versus attempted suicide: similar or different? Arch Suicidal Res 2009 13 2 105 122 10.1080/13811110902834992 19363748

[B8] Hankin BL Abela JRZ Nonsuicidal self-injury in adolescence: prospective rates and risk factors in a 2 ½ year longitudinal study Psychiatry Res 2011 186 1 65 70 10.1016/j.psychres.2010.07.056 20807667 PMC3008214

[B9] Brausch A Guttierrez PM Differences in non-suicidal self-injury and suicide attempts in adolescence J Youth Adolescence 2010 39 233 241 10.1007/s10964-009-9482-0 19941045

[B10] Plener PL Libal G Keller F Fegert JM Muehlenkamp JJ An international comparison of adolescent non-suicidal self-injury (NSSI) and suicide attempts: Germany and the USA Psychol Med 2009 39 1549 1558 10.1017/S0033291708005114 19171079

[B11] Laye-Gindhu A Schonert-Reichl A Nonsuicidal self-harm among community adolescents: understanding the “whats” and “whys” of self-harm J Youth Adolesc 2005 34 447 457 10.1007/s10964-005-7262-z

[B12] Kokkevi A Rotsika V Arapaki A Richardson C Adolescents’ self-reported suicide attempts, self-harm thoughts and their correlates across 17 European countries J Child Psychol Psychiatry 2011 10.1111/j.1469-7610.2011.02457.x 21895649

[B13] Fergusson DM Horwood LJ Ridder EM Beautrais AL Suicidal behaviour in adolescence and subsequent mental health outcomes in young adulthood Psychol Med 2005 35 983 993 10.1017/S0033291704004167 16045065

[B14] Lee A Wong SYS Tsang KK Ho GSM Wong CW Understanding suicidality and correlates among Chinese secondary school students in Hong Kong Health Promot Int 2009 24 2 156 165 10.1093/heapro/dap011 19304991

[B15] Hawton K Rodham K Evans E By Their Own Young Hand. Deliberate Self-Harm and Suicidal Ideas in Adolescence 2006 Jessica Kingsley, London

[B16] Boyd A Golding J MacLeod J Lawlor D Fraser A Henderson J Molloy L Ness A Ring S Davey Smith G Cohort profile: the ‘Children of the 90s’ – the index offspring of the Avon Longitudinal Study of Parents and Children Int J Epidemiol 2012 1 17 10.1093/ije/dys064 22507743 PMC3600618

[B17] Zanarini MC Childhood Interview for DSM-IV Borderline Personality Disorder (CI-BPD) 2003 McLean Hospital, Belmont, MA

[B18] Madge N Hewitt A Hawton K de Wilde EJ Corcoran P Fekete S van Heeringen K De Leo D Ystgaard Y Deliberate self-harm within an international community sample of young people: comparative findings from the child and adolescent self-harm in Europe (CASE) study J Child Psychol Psychiatry 2008 49 6 667 677 10.1111/j.1469-7610.2008.01879.x 18341543

[B19] Paykel ES Myers JK Lindenthal JJ Suicidal feelings in the general population: a prevalence study Br J Psychiatry 1974 124 460 469 10.1192/bjp.124.5.460 4836376

[B20] Donovan J Sanders C Bowling A, Ebrahim S Key issues in the analysis of qualitative data in health services research Handbook of Health Research Methods 2005 Open University Press, Milton Keynes 515 532

[B21] Cox JL Chapman G Murray D Jones P Validation of the Edinburgh Postnatal Depression Scale (EPDS) in nonpostnatal women J Affect Disord 1996 39 3 185 189 10.1016/0165-0327(96)00008-0 8856422

[B22] Cox JL Holden JM Sagovsky R Detection of postnatal depression: development of the 10-item Edinburgh Postnatal Depression Scale Br J Psychiatry 1987 150 782 786 10.1192/bjp.150.6.782 3651732

[B23] Patton GC Olsson C Bond L Toumbourou JW Carlin JB Hemphill SA Catalano RF Predicting female depression across puberty: a two-nation longitudinal study J Am Acad Child Adolesc Psychiatry 2008 47 1424 1432 10.1097/CHI.0b013e3181886ebe 18978636 PMC2981098

[B24] Van Buuren S Boshuizen HC Knook DL Multiple imputation of missing blood pressure covariates in survival analysis Stat Med 1999 18 681 694 10.1002/(SICI)1097-0258(19990330)18:6<681::AID-SIM71>3.0.CO;2-R 10204197

[B25] Royston P Multiple imputation of missing values: Further update of ice, with an emphasis on categorical variables Stata J 2009 9 3 466 477 Retrieved from http://www.stata-journal.com/

[B26] Sterne JAC White IR Carlin JK Spratt M Royston P Kenward MG Wood AM Carpenter JR Multiple imputation for missing data in epidemiological and clinical research: potential and pitfalls BMJ 2009 338 b2393 10.1136/bmj.b2393 19564179 PMC2714692

[B27] White IR Royston P Wood AM Multiple imputation using chained equations: Issues and guidance for practice Stat Med 2011 30 377 399 10.1002/sim.4067 21225900

[B28] Safer DJ Self-reported suicide attempts by adolescents Ann Clin Psychiatry 1997 9 263 269 9511952 10.1023/a:1022364629060

[B29] Gunnell D Shepherd M Evans M Are recent increases in deliberate self-harm associated with changes in socioeconomic conditions? An ecological analysis of patterns of deliberate self-harm in Bristol 1972–3 and 1995–6 Psychol Med 2000 30 5 197 203 10.1017/s0033291799002688 12027054

[B30] Sweeting H Young R West P GHQ increases among Scottish 15 year olds 1987–2006 Soc Psychiatry Psychiatr Epidemiol 2009 44 579 586 10.1007/s00127-008-0462-6 19037574 PMC2693777

[B31] Green H McGinnity A Meltzer H Ford T Goodman R Mental Health of Children and Young People in Great Britain, 2004 2005 Palgrave McMillan, Basingstoke

[B32] Haw C Bergen H Casey D Hawton K Repetition of deliberate self-harm: a study of the characteristics and subsequent deaths in patients presenting to a general hospital according to extent of repetition Suicide Life Threat Behav 2007 37 4 379 396 10.1521/suli.2007.37.4.379 17896879

[B33] Lundh L-G Wangby-Lundh M Bjarehed J Deliberate self-harm and psychological problems in young adolescents: Evidence of a bidirectional relationship in girls Scand J Psychol 2011 Article first published online: 17 MAY 2011 10.1111/j.1467-9450.2011.00894.x 21585393

[B34] Joiner TE Why people die by suicide 2005 Harvard University Press, Cambridge MA

[B35] Martin G Swannell SV Hazell PL Harrison JE Taylor AW Self-injury in Australia: a community survey MJA 2010 193 9 506 510 21034383 10.5694/j.1326-5377.2010.tb04033.x

[B36] Hawton K Harriss L The Changing gender ratio in occurrence of deliberate self-harm across the lifecycle J Crisis Interv Suicide Prev 2008 29 1 4 10 10.1027/0227-5910.29.1.4 18389640

