# Supplementary material for: Adolescent self-harm and suicidal thoughts in the ALSPAC cohort: a self-report survey in England
Source: BMC Psychiatry. 2012 Jun 27;12:69. doi: 10.1186/1471-244X-12-69 (PMC3439325; doi:10.1186/1471-244X-12-69)
Supplement: Additional file 1 — Flow Chart of Cohort Participants. [file 1471-244X-12-69-S1.docx]

**Appendix A**

**Flow Chart of Cohort Participants**

Questionnaire returned

n=4,855

Questionnaire sent

n=9,384

Not singleton or first born of twins, not alive at age one, unknown gender

n=745

Original cohort of pregnancies

n=14,541

Core ALSPAC sample

n=13,796

Lost to follow up, or requested no questionnaire at age 16

n=4,413

Questionnaire not returned

n=4,529

**Data on self-harm available**

**n=4,810**

Data on self-harm missing

n=45
