# Supplementary material for: Adolescent self-harm and suicidal thoughts in the ALSPAC cohort: a self-report survey in England
Source: BMC Psychiatry. 2012 Jun 27;12:69. doi: 10.1186/1471-244X-12-69 (PMC3439325; doi:10.1186/1471-244X-12-69)
Supplement: Additional file 2 — Questions on self-harm. [file 1471-244X-12-69-S2.docx]

## Appendix B

## SECTION XX: DELIBERATE SELF-HARM

| **Life has many ups and downs. Sometimes people feel upset. These feelings can be so bad that people who have them may feel suicidal or want to self-harm. The following questions ask you about your feelings and the feelings of people close to you. We know this is a sensitive subject, but it is important to ask about it now, as it is not uncommon. By finding out about self-harm we can find ways of helping people.** |
| --- |

1. a) Has **anyone** in your family (not including yourself) **ever** hurt themselves on purpose (e.g. by taking an overdose of pills, or by cutting themselves)?

2

1

Yes No **If no, go to question 2**

**If yes,**

b) Who in your family has done this? Please mark **all**  boxes that apply.

1

i) Mum

1

ii) Dad

1

iii) Brother

1

iv) Sister

1

v) Someone else

Please say who:……………………………………………………….

c) Which of these actions best describes what they did? Please mark **all** boxes that apply.

1

i) Swallowed pills or something poisonous

1

ii) Cut themselves

1

iii) Burnt themselves, e.g. with cigarette

1

iv) Something else

Please say what:………………………………………………………….

2. a) Have **any** of your close friends **ever** hurt themselves on purpose?

1

2

Yes No **If no, go to question 3**

**If yes,**

b) Which of these actions best describes what they did? Please mark **all** boxes that apply.

1

i) Swallowed pills or something poisonous

1

ii) Cut themselves

1

iii) Burnt themselves, e.g. with cigarette

1

iv) Something else

Please say what:………………………………………………………….

3. a) Have you **ever** hurt yourself on purpose **in any way** (e.g. by taking an overdose of

pills, or by cutting yourself)?

1

2

Yes No **If no, go to question 6a on page XX**

**If yes,**

1. How many times have you done this in the last year? Please mark **one** box only.

2

1

Once 2-5 times

6-10 times More than

3

4

10 times

c) When was the **last time** you hurt yourself on purpose? Please mark **one** box only.

3

2

1

In the last week More than a week More than a

ago but in the last year year ago

d) The **last time** you hurt yourself on purpose, which of the actions below best

describes what you did? Please mark **all** boxes that apply.

1

i) Swallowed pills or something poisonous

1

ii) Cut yourself

iii) Burnt yourself, e.g. with cigarette

1

iv) Something else

1

Please say what:

………………………………………………………….

e) Do **any** of the following reasons help to explain why you hurt yourself on that occasion? Please mark **all** boxes that apply.

1

i) I wanted to show how desperate I was

feeling

1

1. I wanted to die

1

1. I wanted to punish myself

1

iv) I wanted to frighten someone

1

v) I wanted to get relief from a terrible

state of mind

1

vi) Some other reason

Please say what:………………………………………………………….

f) After you had hurt yourself on that occasion, how did you feel? Please mark **one** box only.

3

2

1

Better than before The same as before Worse than

Before

g) The last time you hurt yourself in any way (e.g. by taking an overdose of pills, or by cutting yourself) did you seek medical help / first aid from any of the following? Please mark **all** boxes that apply.

1

1. GP (family doctor)

1

1. Hospital casuality / emergency department

1

1. Other health professional

Please say what they job was:………………………………………………..

4. On **any** of the occasions when you have hurt yourself on purpose, have you **ever** seriously wanted to kill yourself?

2

1

Yes No

5. a) Have you **ever** tried to get help from someone or somewhere about hurting yourself

on purpose, or about wanting to kill yourself?

2

1

Yes No **If no, go to question 6a**

**If yes,**

b) Who have you been to for help? Please mark **all** boxes that apply.

1

i) Mum or Dad

1

ii) Brother or sister

1

iii) Someone else in your family

1

1. A friend

1

1. A teacher

1

1. A school counsellor

1

1. Peer supporter/mediator at school

1

1. A GP (family doctor)
2. A social worker

1

1. A psychologist or psychiatrist

1

1. A telephone help line

1

1. Somewhere else (e.g. internet, book,

1

magazine, other person, etc.)

Please say what or who:………………………………………………………….

6. a) Have you **ever** felt that life was not worth living?

2

1

Yes No **If no, skip to the end**

**If yes,**

b) When was the **last time** you felt like this? Please mark **one** box only.

3

2

1

In the last week More than a week More than a

ago but in the last year year ago

7. a) Have you **ever** found yourself wishing you were dead and away from it all?

2

1

Yes No **If no, skip to the end**

**If yes,**

b) When was the last time you felt like this? Please mark **one** box only.

3

2

1

In the last week More than a week More than a

ago but in the last year year ago

8. a) Have you **ever** thought of killing yourself, even if you would not really do it?

1

2

Yes No **If no, skip to the end**

**If yes,**

1. When was the last time you felt like this? Please mark **one** box only.

3

2

1

In the last week More than a week More than a

ago but in the last year year ago

9. Have you **ever** made plans to kill yourself?

1

2

Yes No

| ***You can get information and advice relating to any of the questions by contacting the organisations on the enclosed Helpline information sheet.*** |
| --- |
