# Supplementary material for: Adolescent self-harm and suicidal thoughts in the ALSPAC cohort: a self-report survey in England
Source: BMC Psychiatry. 2012 Jun 27;12:69. doi: 10.1186/1471-244X-12-69 (PMC3439325; doi:10.1186/1471-244X-12-69)
Supplement: Additional file 3 — Comparison of relationship between self-harm (SH) and background variables for different imputation samples. [file 1471-244X-12-69-S3.docx]

**Web table: Comparison of relationship between self-harm (SH) and background variables for different imputation samples**

|  |  |  | **Available data**  **(n <= 4,810)** | **Imputation 1**  **(n = 4,810)** | **Imputation 2 (n = 9,383)** | **Imputation 3 (n = 13,796)** |
| --- | --- | --- | --- | --- | --- | --- |
|  |  | SH prevalence | 905 (18.8%) | 905 (18.8%) | 18.4% | 18.7% |
| Gender [ref = males] | Females | OR [95% CI] | 3.42 [2.87, 4.07] | 3.42 [2.87, 4.07] | 3.30 [2.77, 3.92] | 3.24 [2.74, 3.82] |
|  |  | β [MCerror] |  | 1.228 [0.0000] | 1.193 [0.0066] | 1.175 [0.0069] |
|  |  | SE(β) [MCerror] |  | 0.089 [0.0000] | 0.089 [0.0041] | 0.084 [0.0037] |
| Mother’s social class  [ref = prof/managerial] | Intermediate | OR [95% CI] | 0.92 [0.77, 1.08] | 0.94 [0.80, 1.11] | 0.91 [0.77, 1.08] | 0.91 [0.78, 1.06] |
|  |  | β [MCerror] |  | -0.063 [0.0027] | -0.095 [0.0063] | -0.095 [0.0060] |
|  |  | SE(β) [MCerror] |  | 0.084 [0.0006] | 0.086 [0.0030] | 0.078 [0.0042] |
|  | Manual | OR [95% CI] | 1.46 [1.12, 1.90] | 1.40 [1.08, 1.80] | 1.29 [0.99, 1.67] | 1.26 [0.99, 1.59] |
|  |  | β [MCerror] |  | 0.334 [0.0049] | 0.253 [0.0102] | 0.227 [0.0100] |
|  |  | SE(β) [MCerror] |  | 0.129 [0.0013] | 0.132 [0.0051] | 0.120 [0.0053] |
| Mother’s highest educational qualification  [ref = A-level/degree] | O-level | OR [95% CI] | 1.19 [1.01, 1.40] | 1.19 [1.01, 1.40] | 1.18 [1.01, 1.39] | 1.18 [1.01, 1.38] |
|  |  | β [MCerror] |  | 0.170 [0.0013] | 0.167 [0.0054] | 0.167 [0.0058] |
|  |  | SE(β) [MCerror] |  | 0.084 [0.0001] | 0.082 [0.0025] | 0.079 [0.0030] |
|  | < O-level | OR [95% CI] | 1.06 [0.86, 1.29] | 1.07 [0.87, 1.30] | 1.02 [0.82, 1.27] | 1.01 [0.85, 1.19] |
|  |  | β [MCerror] |  | 0.064 [0.0017] | 0.022 [0.0085] | 0.0059 [0.0067] |
|  |  | SE(β) [MCerror] |  | 0.102 [0.0002] | 0.109 [0.0054] | 0.087 [0.0035] |
| Ethnicity  [ref = white] | Non-white | OR [95% CI] | 0.79 [0.53, 1.17] | 0.78 [0.53, 1.16] | 0.91 [0.64, 1.30] | 0.95 [0.66, 1.39] |
|  |  | β [MCerror] |  | -0.228 [0.0029] | -0.094 [0.0129] | -0.046 [0.0162] |
|  |  | SE(β) [MCerror] |  | 0.096 [0.0006] | 0.182 [0.0074] | 0.190 [0.0083] |
| GCSE/GNVQs at grades A*-C  [ref = 5+] | Less than 5 | OR [95% CI] | 1.20 [1.03, 1.34] | 1.26 [1.04, 1.52] | 1.17 [0.97, 1.42] | 1.15 [0.96, 1.38] |
|  |  | β [MCerror] |  | 0.228 [0.0029] | 0.160 [0.0076] | 0.142 [0.0080] |
|  |  | SE(β) [MCerror] |  | 0.096 [0.0006] | 0.095 [0.0040] | 0.092 [0.0047] |
| Short MFQ score aged 16  [ref = <11] | 11 or more | OR [95% CI] | 5.43 [4.60, 6.40] | 5.45 [4.63, 6.43] | 5.38 [4.56, 6.40] | 5.29 [4.45, 6.30] |
|  |  | β [MCerror] |  | 1.696 [0.0011] | 1.684 [0.0060] | 1.667 [0.0073] |
|  |  | SE(β) [MCerror] |  | 0.084 [0.0001] | 0.085 [0.0034] | 0.088 [0.0045] |
| Mother’s EPND score when child aged 11  [ref = <13] | 13 or more | OR [95% CI] | 1.48 [1.17, 1.86] | 1.53 [1.22, 1.92] | 1.53 [1.23, 1.89] | 1.57 [1.27, 1.93] |
|  |  | β [MCerror] |  | 0.425 [0.0044] | 0.424 [0.0081] | 0.449 [0.0089] |
|  |  | SE(β) [MCerror] |  | 0.115 [0.0012] | 0.109 [0.0054] | 0.106 [0.0057] |

Imputation 1: imputing background variables only (the sample for which the outcome was available)

Imputation 2: the sample that received the questionnaire

Imputation 3: the original ALSPAC sample

β's shown are log-odds ratios, i.e. exp(1.228) = 3.42.
